# Association between isolated maternal hypothyroxinemia during the first trimester and adverse pregnancy outcomes in Southern Chinese women: a retrospective study of 7051 cases

**DOI:** 10.1186/s12884-022-05194-w

**Published:** 2022-11-23

**Authors:** Ping Li, Jinhui Cui, Ling Li, Xinjuan Chen, Liping Ouyang, Jianhui Fan, Shuo Lin

**Affiliations:** 1 Department of Obstetrics and Gynaecology, Third Affiliated Hospital of Sun Yat-Sen University, Guangzhou, China; 2 Department of Endocrinology, Third Affiliated Hospital of Sun Yat-Sen University, Guangzhou, China

**Keywords:** Isolated maternal hypothyroxinemia, Adverse pregnancy outcomes, First trimester

## Abstract

**Background:**

The association between isolated maternal hypothyroxinemia (IMH) and adverse pregnancy outcomes is still controversial. This study aimed to evaluate the association between IMH during the first trimester and adverse pregnancy outcomes in southern Chinese women.

**Methods:**

This was a hospital-based, retrospective cohort study. The records of 7051 women, including 1337 IMH women and 5714 euthyroid women who had a singleton pregnancy and accepted routine prenatal service at the Third Affiliated Hospital of Sun Yat-Sen University from January 2015 to September 2018, were extracted from the electronic medical records system in this study. Thyroid functions [thyroid-stimulating hormone (TSH), free thyroxine (fT4) and anti-thyroperoxidase autoantibody (TPO-Ab)] had to be measured before 13 weeks and 6 days of gestation. The chi-square test and multivariate logistic regression analysis were applied to evaluate the association between IMH during the first trimester and adverse pregnancy outcomes.

**Results:**

Prepregnancy obesity [prepregnancy body mass index (preBMI) ≥ 25 kg/m^2^] was found to be more common in the IMH group (11.2% vs. 6.1%) (*P* < 0.05). The prevalence of gestational diabetes mellitus (GDM), postpartum haemorrhage (PPH), macrosomia and large for gestational age (LGA) was higher in the IMH group. However, after using multivariate logistic regression analysis to adjust for confounders (maternal age, educational levels and preBMI), only LGA was shown to be associated with an increased risk in IMH women [adjusted OR: 1.27 (95% CI 1.044–1.566)]. The prevalence of preterm delivery (either < 37 or < 34 weeks), gestational hypertension, preeclampsia, placenta previa, placental abruption, premature rupture of membrane (PROM), intrauterine growth restriction (IUGR), polyhydramnios, stillbirth, small for gestational age (SGA) and low Apgar score did not increase.

**Conclusion:**

IMH during the first trimester did not increase any risk of adverse pregnancy outcomes in southern Chinese women except LGA.

## Background

Thyroid hormones are crucial for normal pregnancy and foetal development. Many changes occur in the physiology of the maternal thyroid gland to maintain an adequate level of thyroid hormones (THs) at each stage of gestation during normal pregnancy [1]. Thyroid disorders might occur if the thyroid gland does not adapt. The most frequent thyroid disorder observed in pregnancy is hypothyroidism [1] and a large number of studies have shown that overt hypothyroidism during pregnancy leads to adverse obstetric complications and affects the health status of the offspring, such as abortion, gestational hypertension, gestational diabetes mellitus (GDM), preeclampsia, placental abruption, preterm delivery, postpartum haemorrhage, and stillbirth [2–4].

In recent years, subclinical thyroid disorders have been the focus. Isolated maternal hypothyroxinemia (IMH), which is defined as a low maternal free thyroxine (fT4) in conjunction with a normal maternal thyrotropin (TSH) level, is a prevalent mild thyroid dysfunction that impacts 1–2% of pregnant women in iodine-sufficient populations [1].Currently, the causes of IMH are still unclear, and obesity, iron deficiency and environmental contaminants have been shown to be associated with IMH [5, 6]. Few studies have investigated the association between IMH and adverse pregnancy outcomes, and the results are still controversial [7]. Some studies have reported that IMH increases the risk of premature delivery, premature rupture of membranes and higher newborn weight [8, 9]. Other studies have reported no increase in the incidences of adverse maternal outcomes and perinatal complications [10–13]. In addition, different studies use different reference ranges for fT4 and TSH, which may significantly influence the comparison between different studies. In this study, we selected a large cohort of women living in an iodine-sufficient coastal city (Guangzhou) in China to investigate whether IMH during the first trimester was associated with adverse pregnancy outcomes.

## Methods

### Subjects

**Fig. 1:**
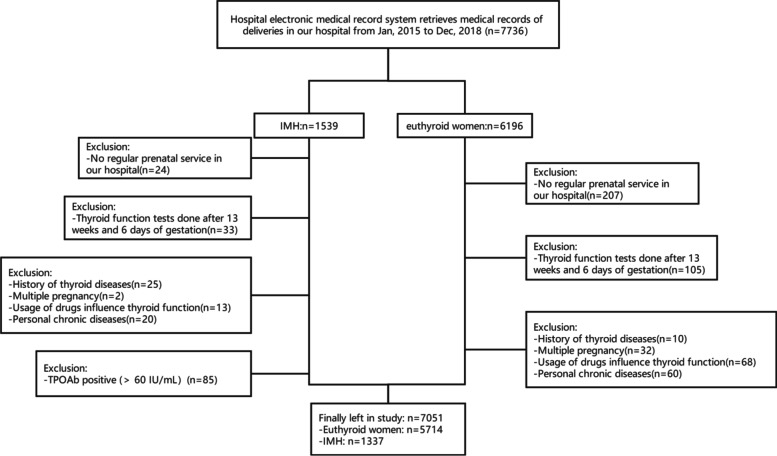
Flow chart showing the women selected for the study. IMH: isolated maternal hypothyroxinemia; TPO-Ab: anti-thyroperoxidase autoantibody

This was a hospital-based, retrospective cohort study. Medical records were retrieved from the electronic medical records system of the Third Affiliated Hospital of Sun Yat-Sen University from January 2015 to December 2018 (*n* = 7736). Women who had a singleton pregnancy, accepted routine prenatal service in our hospital and had thyroid function tests done before 13 weeks and 6 days of gestation were included in this study. Women with a personal history of thyroid diseases, multiple pregnancy, usage of drugs that may influence thyroid function, or chronic diseases (hypertension, cardiac disease, diabetes or autoimmune diseases) were excluded. In the IMH group, women with positive thyroid peroxidase antibody (TPO-Ab) (> 60 IU/mL) were excluded. The study population is shown in a flow chart (Fig. 1). Finally, a total of 7051 pregnant women were recruited for the analysis, including 1337 IMH women and 5714 euthyroid women.

The study was approved by the Human Research Ethics Committee of the Third Affiliated Hospital of Sun Yat-Sen University and conducted in strict accordance with the Declaration of Helsinki.

### Thyroid function tests

Thyroid function, including serum TSH, fT4 and TPOAb levels, was tested during routine prenatal care before 13 weeks and 6 days of gestation in our hospital. Serum samples were obtained in the morning after at least 8 h of fasting and measured at a clinical analysis laboratory by using an automated two-step chemiluminescent immunoassay on an ARCHITECT analyser (Abbott Diagnostics).

### Diagnostic criteria

For the first trimester, the normal range of TSH was 0.1–2.5 mIU/L, according to the 2011 American Thyroid Association (ATA) guidelines [14]. Since there is no recommended normal reference range for fT4, we established a trimester-specific reference range for our hospital according to the standards of the American Institute of Clinical Biochemistry (NACB) [15]. The 2.5th—97.5th percentiles of fT4 among normal pregnant women during the first trimester was 11.90–24.19 pmol/L. The reference range for TPOAb (0–60 IU/mL) was provided by the assay manufacturer, and TPOAb was considered positive if levels were ≥ 60 IU/mL. Women with a low fT4 concentration (< 11.90 pmol/L) in conjunction with a normal TSH concentration (0.1–2.5 mIU/L) were diagnosed as having IMH.

### Data collection

Baseline characteristics of these women were recorded at the patient’s first visit (before 13 weeks and 6 days of gestation) and extracted from the electronic medical records system, which included age, prepregnancy body mass index [BMI, which was calculated by using the formula: BMI = weight (kg)/height (m2)], parity, history of chronic diseases (thyroid disease, hypertension, cardiac disease, diabetes or autoimmune diseases), medications and education levels. Biochemical index was tested at the first visit according to the Clinical practice guideline for prenatal and antenatal care (2011) [16]. Based on previous studies [17, 18], biochemical variables that might have an effect on thyroid function were selected for study, including fasting plasma glucose (FPG) and lipid profiles, including serum total cholesterol (TC), triglycerides (TGs), high-density lipoprotein cholesterol (HDL-C) and low-density lipoprotein cholesterol (LDL-C)]. The test results were also extracted from the electronic medical records system.

### Pregnancy outcome definitions

Obstetric and neonatal adverse outcomes were assessed and documented, including gestational age at delivery, birth method, premature delivery (a live birth before 37 weeks of gestation), GDM (one or more plasma venous glucose values ≥ 5.1 mmol/L at 0 h, ≥ 10.0 mmol/L at 1 h or ≥ 8.5 mmol/L at 2 h after a 2-h 75-g oral glucose tolerance test), gestational hypertension (blood pressure > 140/90 mmHg on at least two occasions more than 6 h apart without evidence of chronic hypertension or significant proteinuria after 20 weeks of gestation), preeclampsia (criteria for gestational hypertension plus significant proteinuria), placenta previa (placenta completely or partially covering the internal cervical os at the time of delivery), placental abruption (premature separation of a normally implanted placenta), premature rupture of membranes (PROM, membrane rupture prior to the onset of labour), intrauterine growth restriction (IUGR, an estimated foetal weight < 10th percentile for gestational age, which was estimated by ultrasonography performed one week before delivery), polyhydramnios (amniotic fluid volume > 8 cm), oligohydramnios (amniotic fluid volume < 2 cm), stillbirth (foetus dies in utero after 20 weeks of gestation) postpartum haemorrhage (PPH, postpartum haemorrhage volume > 500 ml for natural birth or > 1000 ml for caesarean section within 24 h after delivery), small for gestational age (SGA, < 10th percentile of weight in grams for gestational age by gender, according to the Chinese birth weight reference percentiles [19]), large for gestational age (LGA, > 90th percentile of weight in grams for gestational age by gender, according to the Chinese birth weight reference percentiles [19]), and low Apgar score (≤ 7 at 1 or 5 min).

### Statistical analysis

SPSS 23.0 software (IBM Corp. Armonk, NY, USA) was used for data analysis. Data are presented as the mean (SD) for continuous variables, the median (interquartile range) for nonnormally distributed variables and the frequency (percentage) for categorical variables. Clinical characteristics and laboratory parameters between the IMH and euthyroid groups were compared using Student’s t test for quantitative variables and the χ^2^ test for categorical variables. Averse pregnancy outcomes between the two groups were compared using the χ^2^ test.

Multivariate logistic regression analysis was applied to evaluate the correlation between IMH and adverse pregnancy outcomes (enter method was used). Potential confounders included maternal age, preBMI, gravity, parity, education level, FPG, TG, TC, HDL-C and LDL-C, GDM, PROM, gestational hypertension, preeclampsia, placenta previa, placental abruption, polyhydramnios, delivery mode and newborn sex. In the first step, the correlation between adverse pregnancy outcomes and possible predictors was investigated through independent bivariate analysis. After bivariate analysis, correlations with α < 0.1 and those clinically relevant or identified as confounders in previous studies were selected as potential predictors in the regression model. The results are represented as odds ratios (ORs) and 95% confidence intervals (95% CIs). *P* < 0.05 was considered statistically significant.

## Results

A total of 7051 women, including 1337 IMH women and 5714 euthyroid women, were recruited for this study. The characteristics of the participants are shown in Table 1. Compared with euthyroid women, IMH women were older, had more gravidity and were multiparous, had a lower education level, had a higher level of obesity before pregnancy and had a higher newborn weight (all *P* < 0.05). They had rates of vaginal delivery similar to those of euthyroid women (62.8% vs. 63.5%) but a higher rate of caesarean Sect. (37.9% vs. 33.4%) (*P* < 0.05). The postpartum haemorrhage volumes were also higher [320 (250–395) vs. 305 (240–370)] (*P* < 0.05). However, the gestational age at delivery, history of chronic diseases and newborn sex were similar between the two groups. The laboratory parameters of pregnant women with IMH and euthyroids in the first trimester are shown in Table 2. IMH women had higher levels of TSH, TC, TG, HDL-C and LDL-C (all *P* < 0.05).

**Table 1:** Clinical characteristics of pregnant women with IMH and euthyroid status

Characteristics	Euthyroid (*n* = 5714)	IMH (*n* = 1337)	*χ*^*2*^*/Z* value	*P*
Maternal age (years)	29(27–32)	30(27–32)	-4.745	< 0.001
Pregestational BMI (kg/m^2^)			54.619	< 0.001
< 18.5	1205(21.1)	214(16.0)		
18.5–24.9	4162(72.8)	973(72.8)		
≥ 25	347(6.1)	150(11.2)		
Gravidity (%)			31.629	< 0.001
1	2139(37.4)	428(32.0)		
2	2115(37.0)	469(35.1)		
≥ 3	1460(25.6)	440(32.9)		
Parity (%)			7.785	0.005
Nullipara	2909(50.9)	624(46.7)		
Multipara	2805(49.1)	713(53.3)		
Education level (%)			36.003	< 0.001
Junior high school or lower	853(14.9)	259(19.4)		
High school	2142(37.5)	557(41.7)		
Bachelor or higher	2719(47.6)	521(39.0)		
History of chronic diseases (%)			0.014	0.904
Yes	320(5.6)	76(5.7)		
No	5397(94.4)	1261(94.3)		
Gestational age (weeks)	39.29(38.57–40.00)	39.14(38.57–40.00)	-1.776	0.076
Delivery mode (%)			11.997	0.002
Vaginal delivery	3630(63.5)	801(62.8)		
Caesarean section	1907(33.4)	507(37.9)		
Operative vaginal delivery	177(3.1)	29(2.2)		
Postpartum haemorrhage volume (mL)	305(240–370)	320(250–395)	-3.992	< 0.001
Newborn weight (kg)	3.20(2.90–3.40)	3.20(2.95–3.75)	-4.430	< 0.001
Newborn sex (%)			0.908	0.341
Boy	3063(53.6)	736(55.0)		
Girl	2651(46.4)	601(45.0)		

*IMH* isolated maternal hypothyroxinemia, *BMI* body mass index

**Table 2:** Laboratory parameters of pregnant women with IMH and euthyroid status in the first trimester

Characteristics	Euthyroid (*n* = 5714)	IMH (*n* = 1337)	*χ*^*2*^*/Z* value	*P*
fT4 (pmol/L)	13.65(12.87–14.61)	11.27(10.77–11.62)	-57.009	< 0.001
TSH (mU/L)	0.90(0.53–1.37)	1.27(0.88–1.69)	-17.076	< 0.001
FPG (mmol/L)	4.65(4.43–4.88)	4.56(4.35–4.82)	-8.233	< 0.001
TC (mmol/L)	4.43(3.96–4.96)	4.87(4.27–5.57)	-15.210	< 0.001
TG (mmol/L)	1.04(0.83–1.32)	1.35(1.03–1.80)	-18.887	< 0.001
HDL-C (mmol/L)	1.63(1.43–1.86)	1.65(1.44–1.92)	-2.717	0.007
LDL-C (mmol/L)	2.26(1.90–2.64)	2.51(2.09–2.99)	-12.325	< 0.001

*IMH* isolated maternal hypothyroxinemia, *TSH* thyroid stimulating hormone, *fT4*, free thyroxine 4, *FPG* fasting plasma glucose, *TC* total cholesterol, *TG* triglyceride, *HDL-C* high-density lipoprotein cholesterol, *LDL-C* low-density lipoprotein cholesterol

The comparison of adverse pregnancy outcomes between pregnant women with IMH and euthyroid women in the first trimester is presented in Table 3. The prevalence of GDM was higher in the IMH group (23.8% vs. 17.6%, χ^2^ = 27.291, *P* < 0.001). The prevalence of PPH (7.5% vs. 5.8%, χ^2^ = 5.495, *P* = 0.019) was also higher. IMH women were less likely to have oligohydramnios (5.5% vs. 7.5%, χ^2^ = 6.469, *P* = 0.011). For newborns, IMH women had higher chances of delivering heavy foetuses. The prevalences of preterm delivery (either < 37 or < 34 weeks), gestational hypertension, preeclampsia, placenta previa, placental abruption, PROM, IUGR, polyhydramnios, stillbirth, SGA and low Apgar score were not statistically different between the two groups.

**Table 3:** Comparison of adverse pregnancy outcomes between pregnant women with IMH and euthyroid women in the first trimester

Adverse pregnancy outcomes	Euthyroid (*n* = 5714)	IMH (*n* = 1337)	χ^2^	*P*
Mother				
GDM (%)	1005(17.6)	318(23.8)	27.291	< 0.001
Premature delivery				
< 37 weeks	253(4.4)	58(4.3)	0.021	0.886
< 34 weeks	51(0.9)	18(1.3)	2.302	0.129
Gestational hypertension	143(2.5)	30(2.2)	0.304	0.581
Preeclampsia	73(1.3)	21(1.6)	0.705	0.401
Placenta previa	73(1.3)	24(1.8)	2.139	0.144
Placental abruption	62(1.1)	16(1.2)	0.123	0.725
PROM	1246(21.8)	291(21.8)	0.001	0.974
IUGR	102(1.8)	14(1.0)	3.647	0.056
Polyhydramnios	73(1.3)	24(1.8)	2.139	0.144
Oligohydramnios	430(7.5)	74(5.5)	6.469	0.011
Stillbirth	21(0.4)	5(0.4)	0.001	0.972
PPH	330(5.8)	100(7.5)	5.495	0.019
Newborn				
Macrosomia	128(2.2)	49(3.7)	8.988	0.003
SGA	298(5.2)	61(4.8)	0.956	0.328
LGA	661(11.6)	219(16.4)	22.967	< 0.001
Low Apgar score (≤ 7 at 1 or 5 min)	71(1.2)	20(1.5)	0.779	0.677

*IMH* isolated maternal hypothyroxinemia, *GDM* gestational diabetes mellitus, *PROM* premature rupture of membranes, *IUGR* intrauterine growth restriction, *PPH* postpartum haemorrhage, *SGA* small for gestational age, *LGA* large for gestational age

To further study the association between IMH and adverse pregnancy outcomes, we used multivariate logistic regression analysis and adjusted for possible confounders, which are presented in Table 4. This showed that IMH increased the chances of delivering heavy foetuses. The OR and adjusted OR values of LGA and macrosomia were 1.50 (95% CI 1.268–1.768, *P* < 0.001) and 1.66 (95% CI 1.188–2.320, *P* = 0.003), 1.27 (95% CI 1.044–1.566, *P* = 0.017) and 1.46 (95% CI 1.000–2.176, *P* = 0.050), respectively. However, IMH was not associated with GDM, preterm delivery (either < 37 or < 34 weeks), gestational hypertension, preeclampsia, placenta previa, PROM, IUGR, polyhydramnios, stillbirth, SGA or low Apgar score after adjusting for confounders. Interestingly, after adjusting for confounders, IMH was a protective factor against placental abruption 0.612 (95% CI 0.385–0.973, *P* < 0.038).

**Table 4:** Multivariate logistic regression analysis of the association between isolated maternal hypothyroxinemia in the first trimester and adverse pregnancy outcomes

Adverse pregnancy outcomes	Unadjusted model OR (95% CI)	*P*	Adjusted model^1^ 0R (95% CI)	*P*
Mother				
GDM	1.462(1.267–1.687)	< 0.001	1.126(0.947–1.337) ^a^	0.179
Premature delivery				
< 37 weeks	0.979(0.731–1.310)	0.886	0.751(0.533–1.058) ^b^	0.101
< 34 weeks	1.515(0.882–2.602)	0.132	1.170(0.613–2.233) ^b^	0.634
Gestational hypertension	0.894(0.600–1.331)	0.581	0.639(0.407–1.003) ^a^	0.051
Preeclampsia	1.233(0.756–2.010)	0.402	0.740(0.415–1.322) ^a^	0.310
Placenta previa	1.412(0.887–2.249)	0.146	1.242(0.724–2.131) ^a^	0.432
Placental abruption	1.104(0.635–1.919)	0.725	0.612(0.385–0.973) ^c^	0.038
PROM	0.998(0.864–1.152)	0.974	0.971(0.822–1.147) ^b^	0.730
IUGR	0.582(0.332–1.021)	0.059	0.709(0.389–1.293) ^c^	0.262
Oligohydramnios	0.720(0.558–0.928)	0.011	0.781(0.586–1.041) ^c^	0.092
Polyhydramnios	1.412(0.887–2.249)	0.146	1.128(0.646–1.969) ^c^	0.672
Stillbirth	1.018(0.383–2.704)	0.972	0.742(0.254–2.168) ^b^	0.586
PPH	1.319(1.046–1.664)	0.019	1.289(0.983–1.690) ^d^	0.067
Newborn				
Macrosomia	1.660(1.188–2.320)	0.003	1.457(1.000–2.176)^e^	0.050
SGA	0.869(0.655–1.152)	0.329	0.927(0.669–1.283) ^e^	0.646
LGA	1.497(1.268–1.768)	< 0.001	1.270(1.044–1.566) ^e^	0.017
Low Apgar score (≤ 7 at 1 or 5 min)	1.207(0.732–1.989)	0.461	1.207(0.682–2.137) ^d^	0.519

*BMI* body mass index, *GDM* Gestational diabetes mellitus, *PPH* postpartum haemorrhage, *LGA* large for gestational age, *FPG* fasting plasma glucose, *TC* total cholesterol, *TG* triglyceride, *HDL-C* high-density lipoprotein cholesterol, *LDL-C* low-density lipoprotein cholesterol

^a^Adjusted for maternal age, preBMI, gravity, parity, education level, FPG, TG, TC, HDL-C and LDL-C

^b^Adjusted for maternal age, preBMI, gravity, parity, education level, FPG, TG, TC, HDL-C, LDL-C, GDM, gestational hypertension, preeclampsia, placenta previa, placental abruption, polyhydramnios and oligohydramnios

^c^Adjusted for maternal age, preBMI, gravity, parity, education level, FPG, TG, TC, HDL-C, LDL-C, GDM, PROM, gestational hypertension and preeclampsia

^d^Adjusted for maternal age, preBMI, gravity, parity, education level, FPG, TG, TC, HDL-C, LDL-C, GDM, gestational hypertension, preeclampsia, placenta previa, placental abruption, polyhydramnios and delivery mode

^e^Adjusted for maternal age, preBMI, gravity, parity, education level, gestational age, FPG, TG, TC, HDL-C, LDL-C, GDM, and newborn sex

## Discussion

Currently, very few studies have investigated IMH and adverse pregnancy outcomes. The purpose of the present study was to evaluate the association between IMH during the first trimester and adverse pregnancy outcomes in southern Chinese women. We found that the prevalence rates of GDM, PPH, macrosomia and LGA were higher in IMH women. However, after using multivariate logistic regression analysis to adjust for confounders (maternal age, educational levels and preBMI), only LGA was shown to be associated with an increased risk in IMH women (OR: 1.50 (95% CI 1.268–1.768) (*P* < 0.001), adjusted OR: 1.27 (95% CI 1.044–1.566) (*P* = 0.017)). The incidences of preterm delivery (either < 37 or < 34 weeks), gestational hypertension, preeclampsia, placenta previa, PROM, IUGR, polyhydramnios, stillbirth, SGA and low Apgar score did not increase.

The association between IMH and adverse pregnancy outcomes is still controversial. Chen’s [11] study showed that IMH in the first trimester did not increase adverse maternal outcomes or perinatal complications, including placenta previa, placental abruption, premature birth, foetal distress, intrauterine foetal death, low birth weight, GDM, pregnancy-induced hypertension, foetal growth restriction, and PROM. This was similar to our findings. However, they did not include macrosomia or LGA in the analysis. Toloza’s meta-analysis [20] found that IMH was not associated with gestational hypertension or preeclampsia, which was consistent with our findings. Yang’s study [9] found that women with IMH in the first trimester had a higher risk of preterm birth than euthyroid women in either the < 37 or < 34 gestational week groups. A meta-analysis [21] including 35 cohorts also found that among women with IMH, the risk of preterm birth was higher than that in euthyroid women (7.1% vs. 5.0%; absolute risk difference: 2.3%, 95% CI: 0.6%-4.5%; OR: 1.46, 95% CI: 1.12–1.90). However, in these studies, women with TPOAb positivity were not excluded. As antibody positivity may also increase the incidence of preterm birth, this might affect the results of the association between IMH and preterm birth if positive women were not excluded. In our study, we had inconsistent findings, and we did not find that IMH with TPOAb negativity increased the risk of preterm birth. Su et al. [22] analysed 8173 pregnant women in the first trimester and observed that IMH was related to an increased risk of macrosomia but not SGA (OR = 0.81, 95% CI: 0.39–1.66) or LGA (OR = 1.09, 95% CI: 0.74–1.61). In a prospective study by Furnica [23], they compared 55 IMH women and 165 euthyroid women in the first trimester and found a significant increase in macrosomia and a higher caesarean section rate in the IMH group. In a large meta-analysis conducted on this topic by Derakhshan et al. [13] in 2020, 20 cohorts found that IMH was associated with a lower risk of SGA and higher birth weight, and there was an inverse dose‒response association of maternal fT4 with birth weight. These studies all showed that IMH might increase the chances of delivering heavy foetuses. In our study, we also found that IMH was associated with an increased risk of LGA and that the caesarean section rate was higher in the IMH group (39.9% vs. 33.4%, *P* = 0.002). Macrosomia was higher in IMH women (3.7% vs. 2.2%, *P* = 0.003). However, after using the multivariate logistic regression analysis to adjust for confounders (maternal age, educational levels and preBMI), macrosomia was not statistically significant (OR: 1.66, 95% CI: 1.188–2.320, *P* = 0.003; adjusted OR: 1.46, 95% CI: 1.000–2.176, *P* = 0.050). We still thought macrosomia might be a risk factor for IMH. The negative association between fT4 and birth weight was hypothesized to be mediated by an increase in newborn lipid and protein catabolism causing a reduction in caloric availability [13].

The possible reasons for the inconsistent findings of studies might be the variability in the diagnostic criteria of IMH [24], laboratory measurement methods and the differences in the study populations. In most studies, IMH is defined as a fT4 below the 2.5th or 10th percentile with a normal TSH of a given population. Meanwhile, due to changes in human chorionic gonadotropin (HCG), synthesis of thyroxine-binding globulin (TBG) and other factors, fT4 and TSH levels do not remain constant during pregnancy but vary with gestational age [25, 26]. The various studies used different gestational ages at the time of assessment that probably added the variability, and some studies have shown that IMH in different trimesters was associated with different adverse pregnancy outcomes [18, 22, 27]. Differential methods of laboratory measurement as well as ethnic variation may also influence the TSH and fT4 values [28]. At present, the most common uses of manufacturer methods for thyroid function testing include the Roche Elecsys assay, Abbott ARCHITECT i2000 assay and Bayer ADVIA Centaur assay. Therefore, in today’s guidelines, population-based and trimester-specific reference ranges are advised to apply.

Currently, the causes of IMH have not been completely delineated, but some factors have been shown to be associated with IMH, such as obesity, iron deficiency and environmental contaminants [5, 6]. The most common cause of IMH is iodine deficiency [1, 7]. The thyroid will shift its production from T4 to T3 if iodine becomes deficient, which makes T4 decrease. At present, there is a lack of research on the iodine level of pregnant women in Guangzhou. In our study, we also did not measure urinary iodine concentrations (UICs) in pregnant women, which was one of the limitations of our research. However, the subjects lived in the coastal city in southern China (Guangzhou), and the total iodine nutrition level of residents in this province was shown to be in an appropriate range [29]. Overweight can also influence the function of the thyroid gland, usually leading to increased thyrotropin concentrations and changes in the ratio between the hormone triiodothyronine and thyroxine, although within the normal range [30]. In our study, prepregnancy obesity (preBMI ≥ 25 kg/m2) was found to be more common in IMH women, which was also consistent with Han's results [31].

Our study had several strengths. First, it had a large sample size. Second, we comprehensively evaluated the adverse pregnancy outcomes and the relationship with IMH during the first trimester. Furthermore, we adjusted for potential confounders in the analysis of the relationship between the two. Meanwhile, there were also some limitations in our research. First, this was a single-centre study, which might limit its widespread application. Second, in our hospital, no regular thyroid function review was performed in women with IMH. Consequently, the subsequent progression of the disease and whether it had an impact on the adverse pregnancy outcomes were unclear.

## Conclusions

In conclusion, our study elucidated that IMH during the first trimester did not increase any risk of adverse pregnancy outcomes in southern Chinese women except LGA. Whether regular thyroid function review or intervention is needed in women with IMH during pregnancy requires further research.

## Data Availability

All data generated or analysed during this study are available from the corresponding author on reasonable request.
